# A new species and new records of the genus *Alexeter* Förster (Hymenoptera, Ichneumonidae, Ctenopelmatinae) from Beijing with a key to Chinese species

**DOI:** 10.3897/zookeys.858.35012

**Published:** 2019-07-01

**Authors:** Shu-Ping Sun, Tao Wang, Mao-Ling Sheng, Shi-Xiang Zong

**Affiliations:** 1 General Station of Forest and Grassland Pest Management (GSFGPM), National Forestry and Grassland Administration, 58 Huanghe North Street, Shenyang 110034, China General Station of Forest and Grassland Pest Management Shenyang China; 2 Mentougou Forestry Station, Beijing 102300, China Mentougou Forestry Station Beijing China; 3 College of Forestry, Beijing Forestry University, Beijing 100083, China Beijing Forestry University Beijing China

**Keywords:** Mesoleiini, host, taxonomy

## Abstract

A new species, *Alexeterbeijingensis* Sheng, **sp. nov.**, and two new records for China, *A.angularis* (Uchida, 1952) and *A.shakojiensis* Uchida, 1930, collected in Mentougou, Beijing, belonging to the tribe Mesoleiini of the subfamily Ctenopelmatinae (Hymenoptera, Ichneumonidae), are reported. A key to the six species of *Alexeter* known from China is given.

## Introduction

*Alexeter* Förster, 1869, belonging to the tribe Mesoleiini of the subfamily Ctenopelmatinae (Hymenoptera: Ichneumonidae), comprises 32 species (Yu et al. 2016), of which eleven are from the Eastern Palaearctic Region (seven of them also occur in the Western Palaearctic region) (Šedivý 1971, Uchida 1930, 1952, Yu et al. 2016), 18 from Western Palaearctic (Aubert 1998, Hinz 1996, Heinrich 1949, 1953, Meyer 1936), three from the Neotropical (Gauld et al. 1997), and seven from the Nearctic Region (Yu et al. 2016).

To date, three species are known from China (Chao 1976, Luo et al. 2019, Roman 1936). *Alexeterclavator* (Müller, 1776) was mentioned by Chao (1976) from Gansu, NW China, *A.multicolor* (Gravenhorst, 1829) from Jiangxi and Henan, S China, was reported by Luo et al. (2019), *A.segmentarius* (Fabricius, 1787) was reported from Gansu by Roman (1936) and Chao (1976).

The known hosts of *Alexeter* are sawflies, belonging to Diprionidae and Tenthredinidae (Aubert 1998, 2000, Constantineanu and Istrate 1973, Gauld et al.1997, Hinz 1961, 1996, Yu et al. 2016).

In this paper we deal with all *Alexeter* species from China, including the description of a new species and a key to all known species from the country.

## Materials and methods

Specimens were collected by interception traps (IT) (Li et al. 2012) in the forest of Mentougou, Beijing, P.R. China. The forest of Mentougou is composed of mixed deciduous angiosperms and evergreen conifers (Zong et al. 2013). Images were taken using a Leica M205A stereomicroscope with LAS Montage MultiFocus. Morphological terminology is mostly based on Gauld (1991).

The specimens of *A.clavator* (Müller, 1776), *A.coxalis* (Brischke, 1871), *A.multicolor* (Gravenhorst, 1829), *A.nebulator* (Thunberg, 1822), *A.niger* (Gravenhorst, 1829), *A.rapinator* (Gravenhorst, 1829), *A.segmentarius* (Fabricius, 1787) provided by Dr. Gavin Broad (The Department of Life Sciences, the Natural History Museum, London, UK) (**NHMUK**), were examined. The photos of the types, described by Uchida and deposited in Hokkaido University Museum, Hokkaido University, Japan, taken by Dr. Kyohei Watanabe (Kanagawa Prefectural Museum of Natural History, Odawara, Japan) (**KPMNH**), were examined and compared to the new species by the corresponding author.

Type specimens are deposited in the Insect Museum, General Station of Forest and Grassland Pest Management (**GSFGPM**), National Forestry and Grassland Administration, People’s Republic of China.

## *Alexeter* Förster, 1869

**Type species.***Mesoleptusruficornis* Gravenhorst, 1829 (= *segmentarius* Fabricius, 1787).

**Diagnosis.** (Förster 1869, Gauld et al. 1997, Townes 1970). Clypeus separated from face, apical margin blunt, weakly concave or centrally truncate. Occipital carina dorsally complete, reaching to hypostomal carina distinctly above base of mandible. Epomia absent. Notaulus usually long and sharp. Mesopleuron with very fine to large punctures. Median longitudinal carina of propodeum usually distinct and complete, pleural carina complete. Fore wing vein 1cu-a opposite or distal of 1-M. Areolet present, or 3rs-m absent (Gauld et al. 1997). Hind wing vein 1-cu longer than cu-a. First tergite slender, median dorsal carina absent, or vestigial or indistinct. Glymma present. Second tergite with fine, weak, or indistinct punctures. Ovipositor short, with a distinct dorsal subapical notch.

### Key to species of *Alexeter* known from China

**Table:** 

1	Body brown to reddish brown. Postocellar line 1.3 times as long as ocular-ocellar line. Malar space approximately 0.2 times as long as basal width of mandible. Median longitudinal carina of propodeum distinct and complete. Third tergite approximately 1.3 times as long as maximum width.	***A.clavator* (Müller)**
–	Body, at least mesosoma, black. Other characters variable.	**2**
2	Median longitudinal carinae of propodeum complete, area superomedia distinctly constricted (Fig. 7) or expanded medially. Metasomal tergites black, or almost entirely black.	**3**
–	Median longitudinal carinae of propodeum almost parallel (Fig. 14), or divergent posteriorly, or incomplete (Fig. 13). Basal or median tergites, or all tergites brown to red-brown.	**5**
3	Median longitudinal carinae of propodeum distinctly expanded medially (Fig. 12). Tegula, scutellum and postscutellum pale yellow.	***A.multicolor* (Gravenhorst)**
–	Median longitudinal carinae of propodeum distinctly constricted medially. Tegula, scutellum and postscutellum black.	**4**
4	Fore wing vein 2m-cu connecting to areolet basad of its posterior angle. Ovipositor sheath narrowed backwardly. Middle tarsus and hind leg entirely black.	***A.angularis* (Uchida)**
–	Fore wing vein 2m-cu connecting to 4-M slightly distal of posterior angle of areolet. Ovipositor sheath parallel-sided. Subbasal portion of hind tibia widely white. Middle tarsus with at least median portion white or yellowish brown. Hind tarsomeres 2–4 and basal half of 5 white.	***A.beijingensis* sp. nov.**
5	Median longitudinal carinae of propodeum almost parallel (Fig. 14). Third tergite distinctly longer than its apical width. Hind femur and second and subsequent tergites reddish brown. Ovipositor sheath whitish yellow.	***A.shakojiensis* Uchida**
–	Median longitudinal carinae of propodeum divergent backwardly or incomplete (Fig. 13). Third tergite shorter than its apical width. Hind femur, fifth and subsequent tergites black. Ovipositor sheath black.	***A.segmentarius* (Fabricius)**

### Alexeter beijingensis

Taxon classificationAnimaliaHymenopteraIchneumonidae

Sheng sp. nov.

http://zoobank.org/A36502D6-F805-4493-AFA6-4EAC2B14A464

Figs 1 , 2 , 3 , 4 , 5 , 6 , 7 , 8 , 9 , 10 , 11

#### Etymology.

The name of the new species is derived from the type locality.

#### Material examined.

Holotype female, Mentougou, Beijing, 20 August 2004, leg. Tao Wang and Shi-Xiang Zong (GSFGPM).

#### Diagnosis.

Apical portion of clypeus shiny, apical margin weakly and evenly concave. Outer profiles of middle and hind tibiae with distinct spines. Propodeum (Fig. 7) shagreened, area between median longitudinal carinae shiny, almost smooth; posterolateral portion with long and dense grey setae. Head, mesosoma and metasoma almost entirely black. Subbasal portions of all tibiae and median bands of middle and hind tarsi white.

#### Description.

Female. Body length approximately 11.5 mm. Fore wing length 10.0 mm.

#### Head.

Inner margins of eyes weakly indented opposite antennal sockets. Face (Fig. 2) 1.3 times as wide as long, uppermedian portion slightly convex; shagreened, with dense fine indistinct punctures; upper margin with a small median smooth tubercle. Clypeus (Fig. 2) transversely convex medially; basal portion shagreened, with indistinct short transverse wrinkle; apical portion smooth, shiny; apical margin weakly and evenly concave. Mandible with dense dark grey setae, lower tooth slightly longer than upper tooth. Malar space 0.5 times as long as basal mandibular width. Gena almost evenly convergent backward, in dorsal view approximately 0.8 times as long as width of eye. Vertex (Fig. 3) and frons with sculpture as that of face. Postocellar line 0.5 times as long as ocular-ocellar line. Antenna with 50 flagellomeres; ratios of lengths from first to fifth flagellomeres: 4.8:2.2:2.0:1.9:1.7; ultimate flagellomere twice as long as penultimate flagellomere. Occipital carina complete.

**Figure 1.**
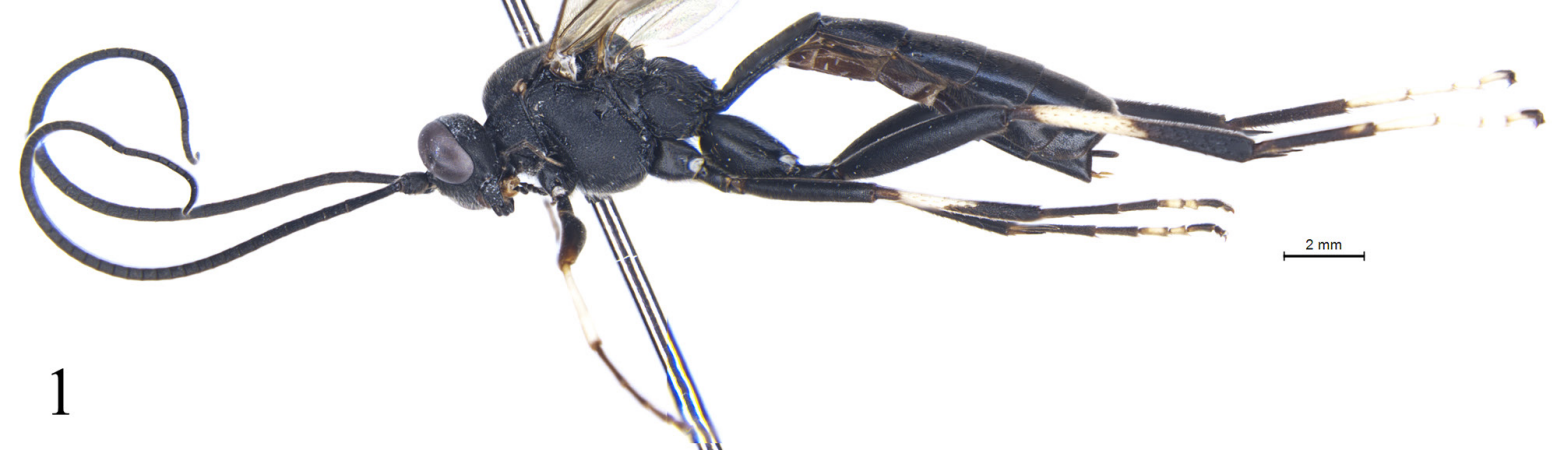
*Alexeterbeijingensis* sp. nov. Holotype. Female. Habitus (without wings), lateral view.

**Figure 2.**
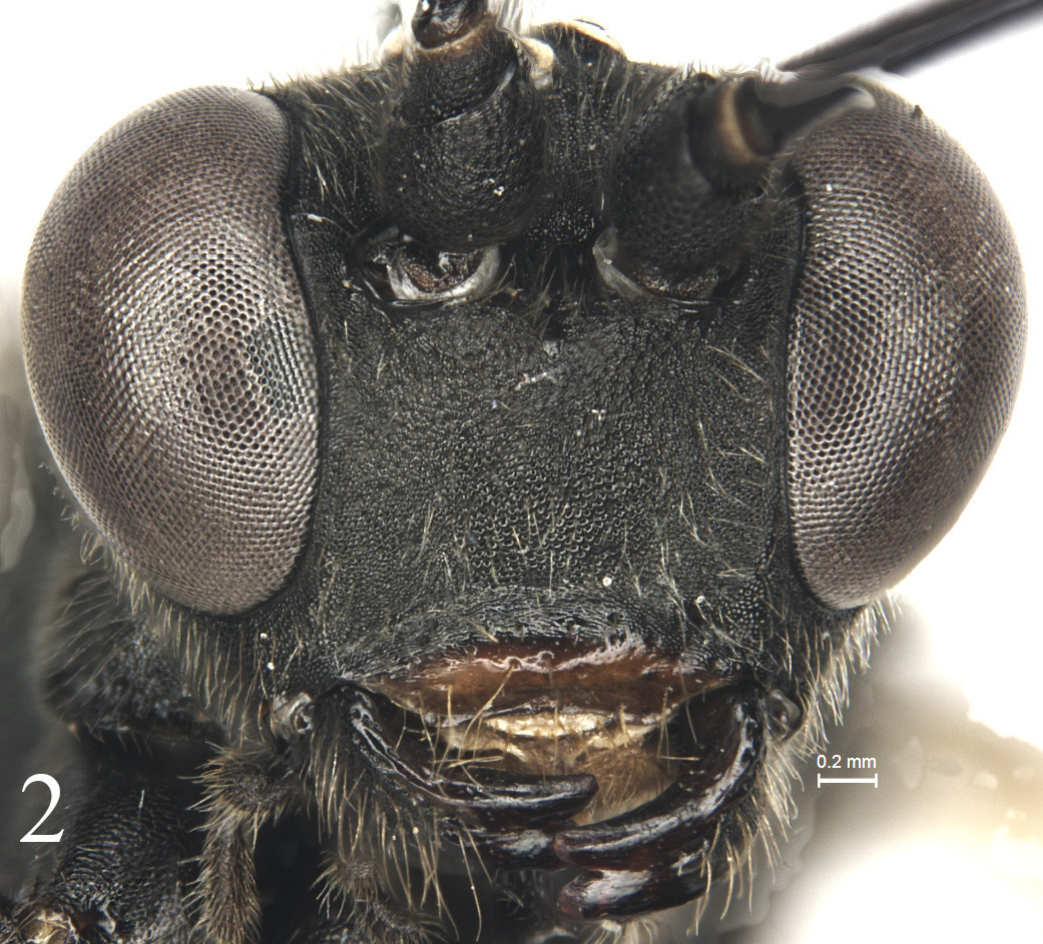
*Alexeterbeijingensis* sp. nov. Holotype. Female. Head, anterior view.

**Figure 3.**
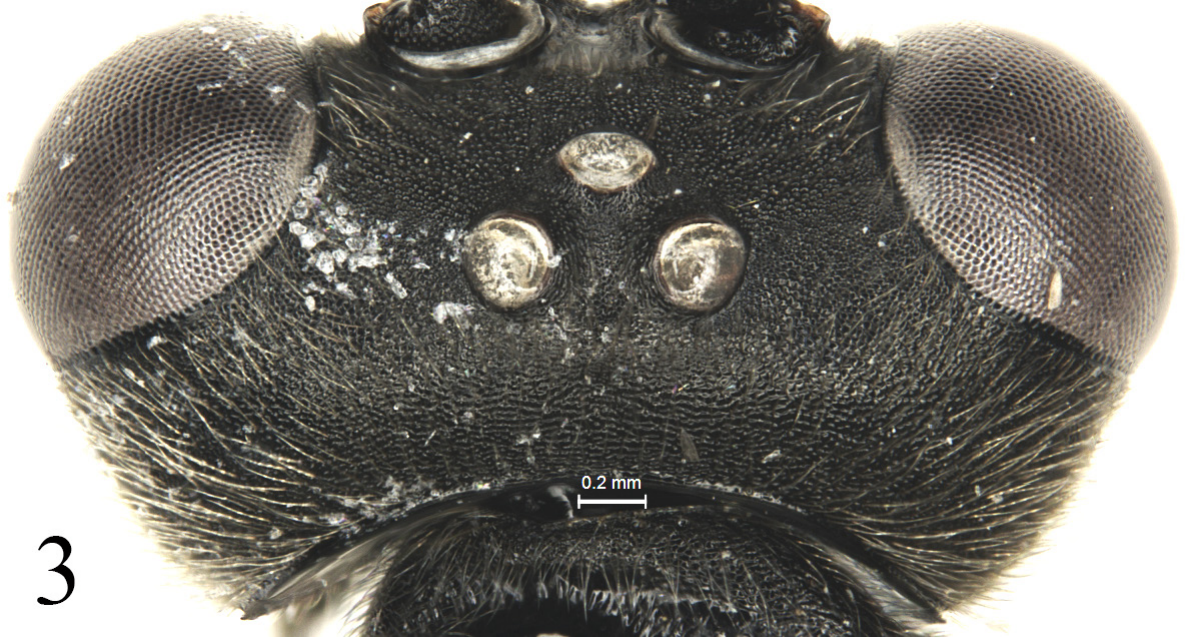
*Alexeterbeijingensis* sp. nov. Holotype. Female. Head, dorsal view.

#### Mesosoma.

**Figure 4.**
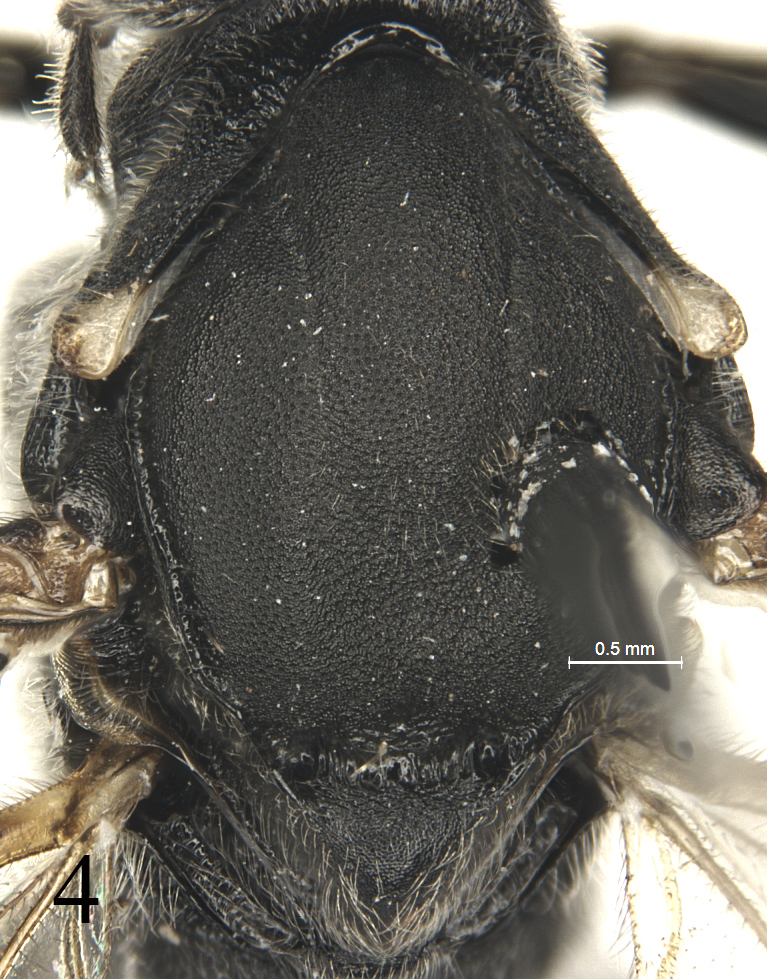
*Alexeterbeijingensis* sp. nov. Holotype. Female. Mesoscutum and scutellum, dorsal view.

**Figure 5.**
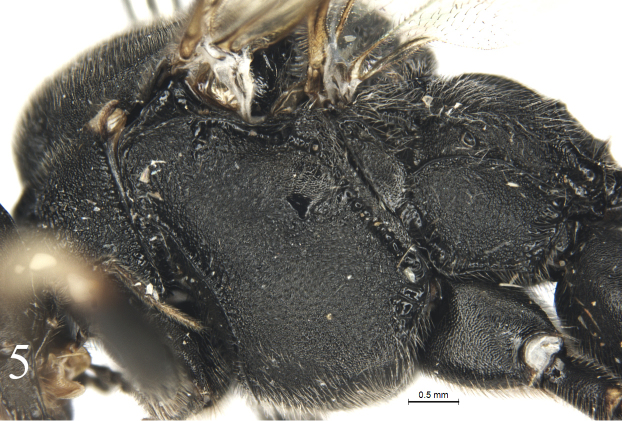
*Alexeterbeijingensis* sp. nov. Holotype. Female. Mesosoma, lateral view.

**Figure 6.**
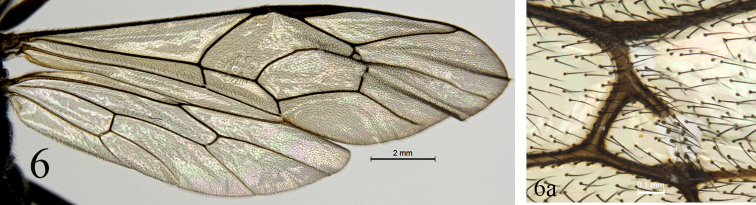
*Alexeterbeijingensis* sp. nov. Holotype. Female **6** wings **6a** areolet.

**Figure 7.**
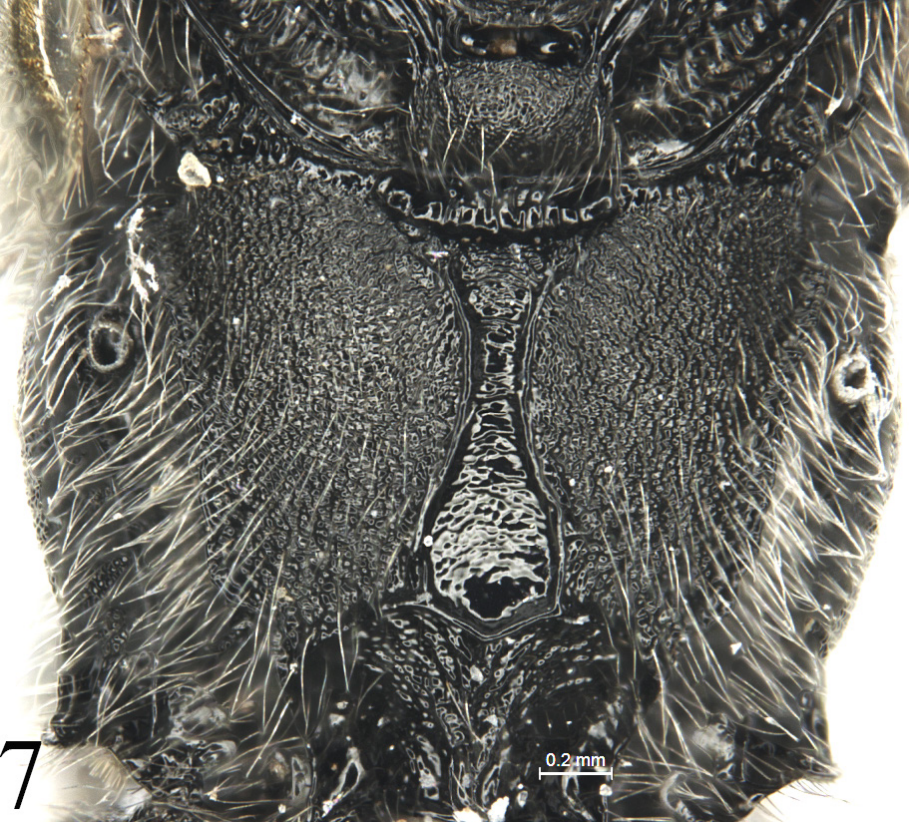
*Alexeterbeijingensis* sp. nov. Holotype. Female. Propodeum, dorsal view.

**Figure 8.**
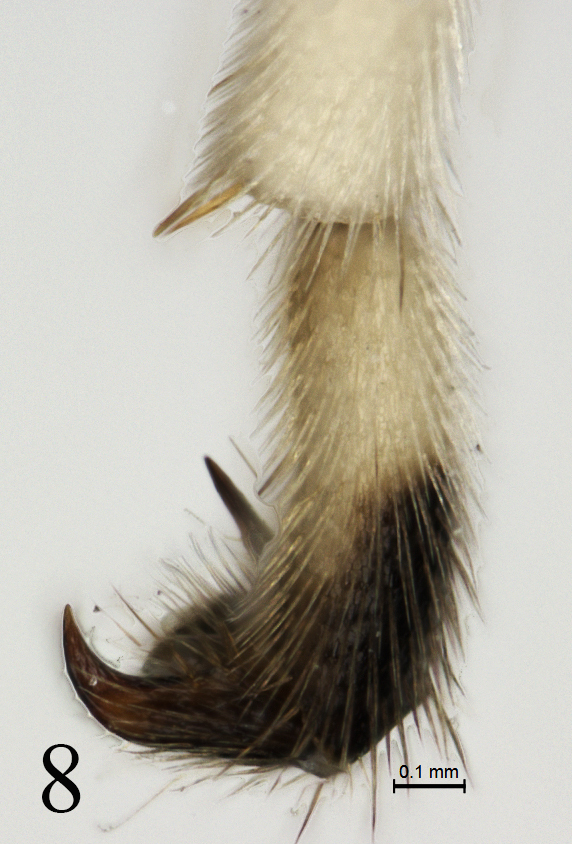
*Alexeterbeijingensis* sp. nov. Holotype. Female. Apex of hind tarsus with claw, lateral view.

Pronotum shagreened, upper portion with dense fine transverse wrinkles; hind margin of lateral concavity with short transverse oblique wrinkles. Epomia indistinct. Mesoscutum (Fig. 4) finely shagreened, with relatively sparse shallow punctures, distance between punctures mostly subequal to one diameter of puncture. Notaulus weak, present on front half of mesoscutum. Scutoscutellar groove with dense longitudinal wrinkles. Scutellum and postscutellum finely shagreened. Mesopleuron (Fig. 5) with sculpture almost as that of mesoscutum, with indistinct fine punctures. Speculum very small, upper portion shagreened. Mesopleural fovea vestigial. Upper end of epicnemial carina almost reaching anterior margin of mesopleuron, at level of upper 0.6 of pronotum. Metapleuron almost flat, with sculpture as that of mesopleuron; posterior margin with short transverse wrinkles. Submetapleural carina distinct, complete. Wings (Fig. 6) slightly infuscate. Fore wing with vein 1cu-a distal to 1-M by 0.3 times length of 1cu-a. Areolet triangular, with long petiole, 0.4 times length of its height. 2m-cu slightly reclivous, connecting to posterior angle of areolet. Hind wing vein 1-cu 1.5 times as long as cu-a. Outer profiles of middle and hind tibiae with relative dense spines. Ratio of length of hind tarsomeres from first to fifth is 4.0:2.0:1.5:0.8:1.0. Tarsal claws simple, hind claw strongly thick and curved (Fig. 8). Propodeum (Fig. 7) with distinct posterior transverse and strong complete median longitudinal carinae, latter strongly constricted medially. Area between median longitudinal carinae shiny, with indistinct, irregular transverse oblique fine striae. Area petiolaris with irregular longitudinal wrinkles. Remainder with sculpture as that of mesopleuron. Posterolateral portion with long dense grey hairs. Propodeal spiracle circular.

#### Metasoma

**Figures 9.**
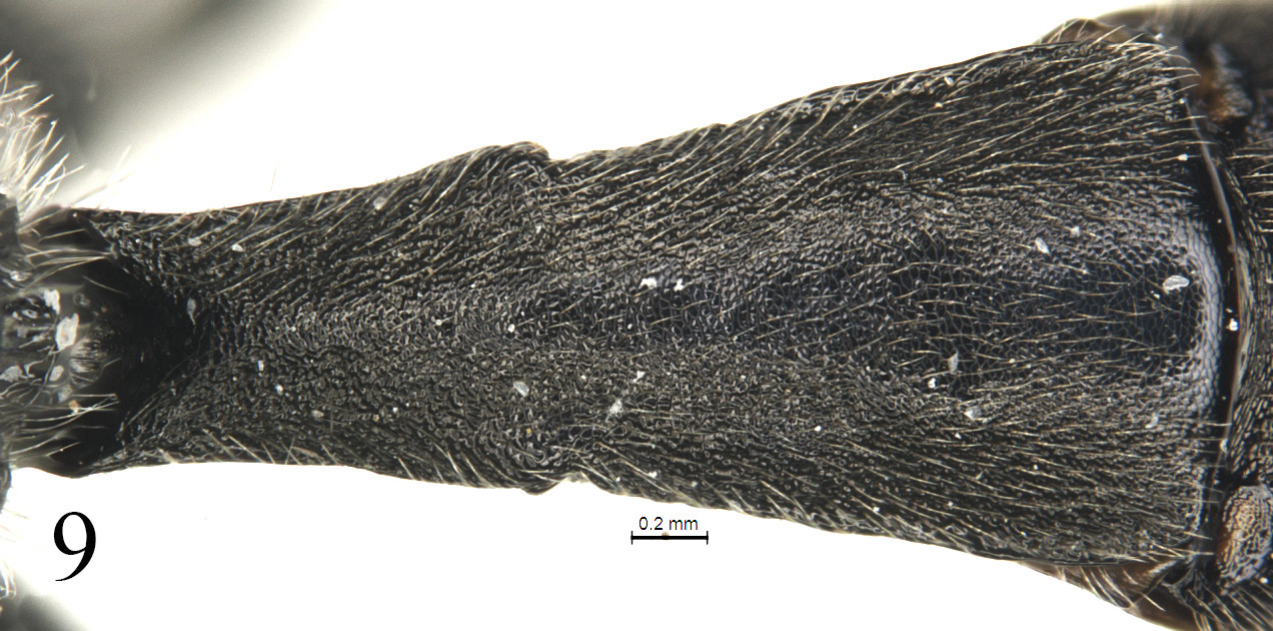
*Alexeterbeijingensis* sp. nov. Holotype. Female. First tergite, dorsal view.

**Figures 10.**
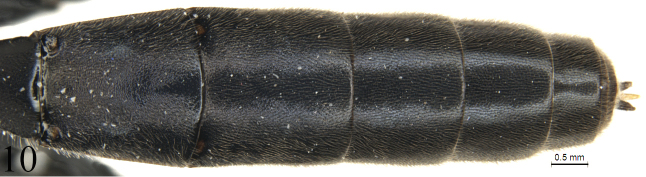
*Alexeterbeijingensis* sp. nov. Holotype. Female. Tergites 2-8, dorsal view.

**Figures 11.**
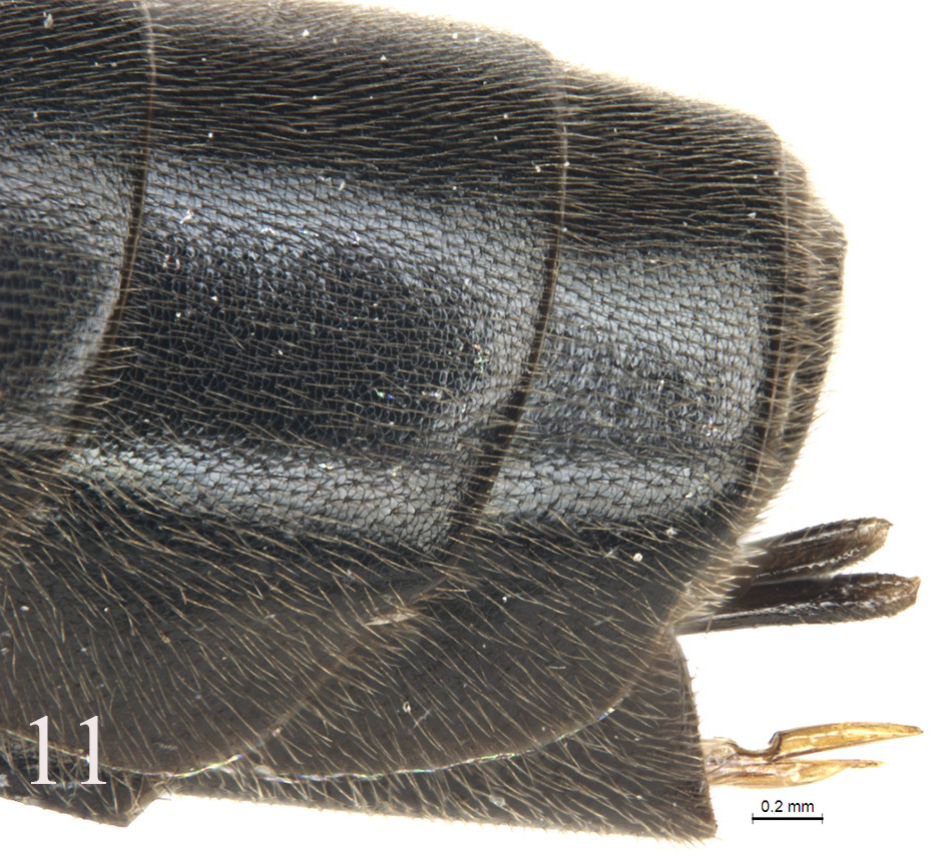
*Alexeterbeijingensis* sp. nov. Holotype. Female. Apex of metasoma with ovipositor, lateral view.

(Figs 9–11). Tergites shagreened. First tergite (Fig. 9) 2.4 times as long as apical width; median dorsal carina absent; dorsolateral carina indistinct, almost absent; spiracle distinctly convex, located slightly before mid of the tergite. Second tergite (Fig. 10) 1.1 times as long as apical width. Lateral margins of tergites 3 and 5, in dorsal view, almost parallel. Third tergite 1.1 times as long as apical width. Fourth tergite 0.8 times as long as apical width. Ovipositor sheath 0.5 times apical depth of metasoma. Ovipositor (Fig. 11) tapered from base to apex, with a large, deep, almost quadrangular notch.

#### Coloration

(Fig. 1). Black, except for the following. Apical half of clypeus, anterior side and apical portion of fore femur red brown. Dorsoposterior portion of pronotum, fore tibia, basal halves of middle and hind tibiae except bases, mid tarsomeres 3 and 4, and hind tarsomeres 2–5 white. Apicomedian portion of scutellum yellowish white. Pterostigma and wing veins brownish black.

#### Comparative diagnosis.

The new species is similar to *A.niger* (Gravenhorst, 1829) in coloration: mesosoma and metasoma black; basal and apical portions of hind tibiae black, median portions white or yellowish white, but can be distinguished from the latter by the following combinations of characters: median longitudinal carina of propodeum complete, strong (absent or indistinct in *A.niger*); fore wing vein 2m-cu connecting to posterior angle of areolet (basad in *A.niger*); antenna, face and tegula black (antenna yellow-brown, face and tegula yellow in *A.niger*).

### Alexeter angularis

Taxon classificationAnimaliaHymenopteraIchneumonidae

(Uchida, 1952)

#### Material examined.

CHINA: 1 female, Mentougou, Beijing, 29 September 2009, leg. Tao Wang.

#### Distribution.

China, Japan. New record for China.

### Alexeter clavator

Taxon classificationAnimaliaHymenopteraIchneumonidae

(Müller, 1776)

#### Material examined.

CHINA: 105 females, 121 males, Mt. Liupanshan, Ningxia Hui Autonomous Region, 7 July to 19 September 2005, IT. 1 female, Qinling, Shaanxi province, 5 July 2017, leg. Tao Li.

#### Distribution.

China: Gansu, Ningxia, Shaanxi; Finland; Germany; Netherlands; Sweden; Switzerland.

### Alexeter multicolor

Taxon classificationAnimaliaHymenopteraIchneumonidae

(Gravenhorst, 1829)

Fig. 12

#### Material examined.

CHINA: 1 female, 2 males, Tianzhu, Guizhou province, April 1996, leg. Yi-Han Li. 1 female, Jiulianshan, Jiangxi province, 5 August 2012, IT. 2 males, Wugongshan, 580 m, Jiangxi province, 16 May 2016, leg. Yu Yao. 1 male, Baotianman National Natural Reserve, 1300–1500 m, Henan province, 12 July 1998, leg. Mao-Ling Sheng.

**Figure 12.**
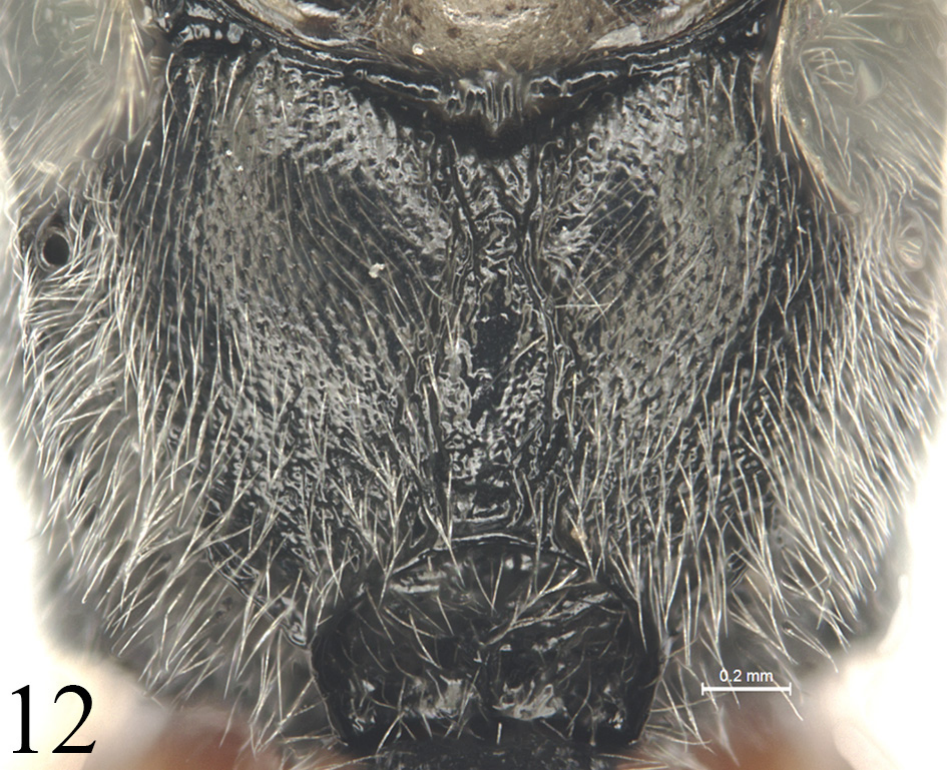
*Alexetermulticolor* (Gravenhorst, 1829). Propodeum, dorsal view.

#### Distribution.

China: Guizhou, Henan, Jiangxi; Europe (Yu et al. 2016).

### Alexeter segmentarius

Taxon classificationAnimaliaHymenopteraIchneumonidae

(Fabricius, 1787)

Fig. 13

#### Material examined.

CHINA: 1 male, Zhenfengshan, Helong, Jilin province, 2 August 1982, leg. Bing-Zong Ren.

**Figure 13.**
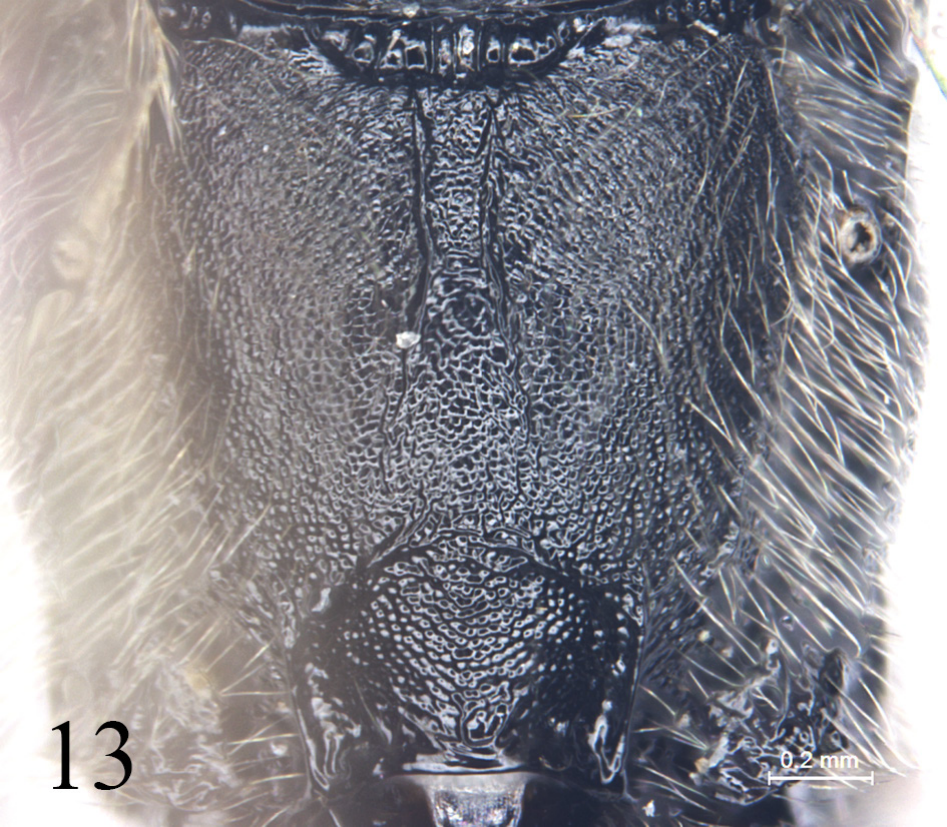
*Alexetersegmentarius* (Fabricius, 1787). Propodeum, dorsal view.

#### Distribution.

China: Gansu, Jilin; Mongolia, Russia, Europe (Yu et al. 2016)

### Alexeter shakojiensis

Taxon classificationAnimaliaHymenopteraIchneumonidae

Uchida, 1930

Fig. 14

#### Material examined.

CHINA: 6 females, Mentougou, Beijing, 29 August to 22 September 2008, leg. Tao Wang. 1 female, 1 male, Mentougou, Beijing, 4 August to 8 September 2009, leg. Tao Wang. 1 male, Yanqing, Beijing, 12 June 2012, leg. Shi-Xiang Zong.

**Figure 14.**
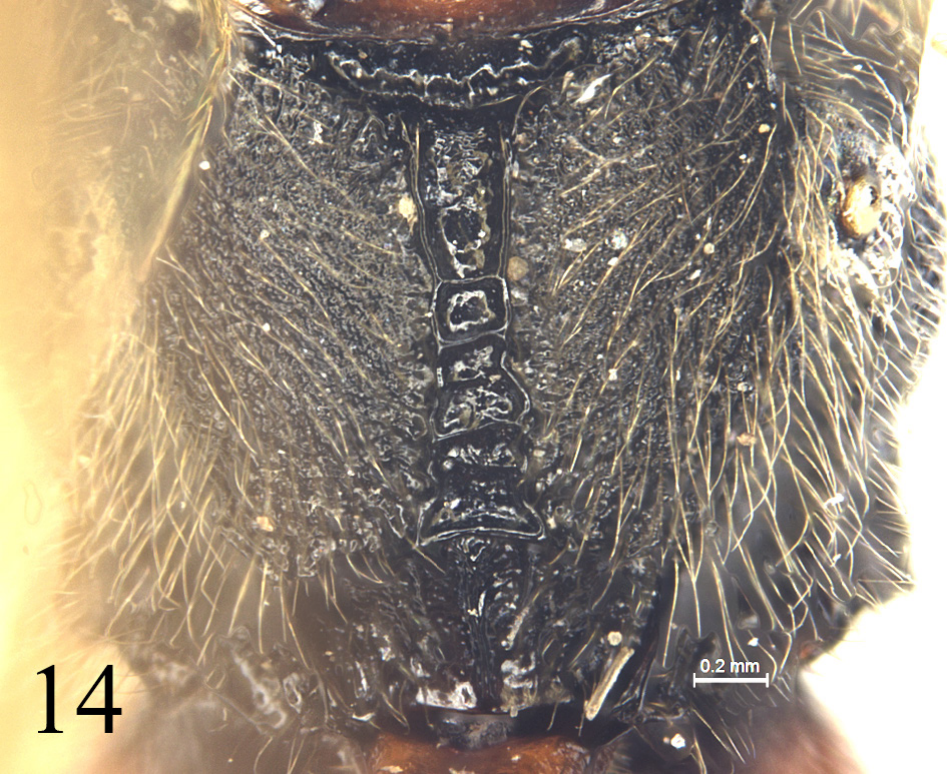
*Alexetershakojiensis*Uchida 1930. Propodeum, dorsal view.

#### Distribution.

China, Korea. New record for China.

## Supplementary Material

XML Treatment for Alexeter beijingensis

XML Treatment for Alexeter angularis

XML Treatment for Alexeter clavator

XML Treatment for Alexeter multicolor

XML Treatment for Alexeter segmentarius

XML Treatment for Alexeter shakojiensis
